# Role construction and boundaries in interprofessional primary health care teams: a qualitative study

**DOI:** 10.1186/1472-6963-13-486

**Published:** 2013-11-24

**Authors:** Kate MacNaughton, Samia Chreim, Ivy Lynn Bourgeault

**Affiliations:** 1 Telfer School of Management, University of Ottawa, Ottawa, Canada; 2 Interdisciplinary School of Health Sciences, University of Ottawa, Ottawa, Canada

**Keywords:** Role boundaries, Interprofessional collaboration, Influences on role construction, Comparative case study

## Abstract

**Background:**

The move towards enhancing teamwork and interprofessional collaboration in health care raises issues regarding the management of professional boundaries and the relationship among health care providers. This qualitative study explores how roles are constructed within interprofessional health care teams. It focuses on elucidating the different types of role boundaries, the influences on role construction and the implications for professionals and patients.

**Methods:**

A comparative case study was conducted to examine the dynamics of role construction on two interprofessional primary health care teams. The data collection included interviews and non-participant observation of team meetings. Thematic content analysis was used to code and analyze the data and a conceptual model was developed to represent the emergent findings.

**Results:**

The findings indicate that role boundaries can be organized around interprofessional interactions (giving rise to *autonomous or collaborative roles*) as well as the distribution of tasks (giving rise to *interchangeable or differentiated roles*). Different influences on role construction were identified. They are categorized as structural (characteristics of the workplace), interpersonal (dynamics between team members such as trust and leadership) and individual dynamics (personal attributes). The implications of role construction were found to include professional satisfaction and more favourable wait times for patients. A model that integrates these different elements was developed.

**Conclusions:**

Based on the results of this study, we argue that autonomy may be an important element of interprofessional team functioning. Counter-intuitive as this may sound, we found that empowering team members to develop autonomy can enhance collaborative interactions. We also argue that while more interchangeable roles could help to lessen the workloads of team members, they could also increase the potential for power struggles because the roles of various professions would become less differentiated. We consider the conceptual and practical implications of our findings and we address the transferability of our model to other interprofessional teams.

## Background

Interprofessional collaboration is increasingly being promoted as a mechanism to respond to the challenges of the health care system by reducing costs, improving quality of care, and improving staff retention and job satisfaction [1]. Accompanying this trend towards teamwork are issues around the management of professional boundaries and the relationship among health care providers [2]. For example, Byrnes et al. observe that placing health care providers of different professional backgrounds on a team does not mean that they will have the knowledge and skills necessary to work together and collaborate [1]. Similarly, D’Amour et al. [3] state that “one of the major challenges facing interprofessional practice is how professional territories are carved out and distributed within a complex system.” The current emphasis on interprofessional collaboration, and the necessity of synergizing professional roles, suggest the need to better understand how roles are constructed on interprofessional health care teams.

A variety of studies have made contributions to the body of knowledge on health care teams, yet the diversity of, and the numerous dynamics found in these groups means that they cannot be accounted for by a ‘one-size-fits-all’ framework. In their review of team research, Mathieu et al. [4] call for researchers to “ensure that we are capturing and embracing the complexities of current team arrangements and seeking to better understand them rather than to fit them into our current frameworks.” Accordingly, this study aims to understand how roles are constructed within interprofessional health care teams in primary care. Our focus is on the types of role boundaries and the influences on role construction. Through the thematic content analysis of interview and observation data obtained from two primary health care teams, we have generated a model to reflect the elements of role construction.

Primary health care has a mandate to provide services delivered by a collaborative team of professionals while emphasizing the quality of care and health status of patients [5]. According to Saba et al. [6] newer models of primary care necessitate a shift in practice, from the historical system of a lone physician to that of a high functioning primary health care team. In Canada, different primary care models offer an aggregation of health services within one organization (e.g. Community Health Centre, Family Health Team). As with team-based models in other settings, many challenges are encountered when trying to provide care across a diverse set of professionals. Difficulties include coordinating the roles of the different professionals to create a cohesive and complementary set of services for the benefit of the patients and the team members [7]; and overcoming a lack of trust and respect between team members [8]. These challenges are often experienced in micro-sites which are arenas for ongoing boundary work [9] through which professional roles are negotiated and constructed.

Professional role construction can be defined as the creation and negotiation of *taskwork*, where taskwork refers to the functions that individuals must perform to accomplish the team’s task [10]. This concept is similar to Forsyth’s outline of task roles within a group, where task roles are aimed at the completion of a group’s goals and at supporting team members’ efforts to do the same [11].

In order to examine role construction, it is pertinent to consider literature on role boundaries. The roles performed by different members of interprofessional teams are subject to professional boundaries [12,13]. These boundaries have been described as contested spheres of practice produced by a ‘labour of division’ [12]. For example, Abbott points out that professions cultivate unique knowledge systems in order to maintain their ‘exclusive property’ and sphere of influence [14]. However, Bourgeault and Mulvale have highlighted the efforts of regulatory agencies to break down exclusive professional boundaries on health teams given that overlapping scopes of practice allow teams to be more responsive to changing conditions [12]. This stream of research has pointed to macro influences on role construction. Role boundaries can also be negotiated and constructed in micro sites where they are shaped by local forces and the interactions among members [15,16]. Chreim et al. [15] point to the importance of meanings, actions and interactions of professionals in organizational settings for an understanding of role construction. In this study, we focus on the construction of role boundaries in micro-sites but acknowledge that this phenomenon takes place within macro-level constraints.

As part of our focus on role construction at the team level, we are examining boundaries that form around team member interactions and around role distribution between professions. In team studies, the intensity of interactions between team members is frequently characterized using the terms ‘autonomy’ and ‘collaboration’. Collaboration is an interpersonal process that entails joint involvement in intellectual activities [17] whereas autonomy suggests independent and self-determined practice [18]. Although these two concepts may appear by definition to be opposed to each other, in practice professional work involves both independent and interdependent elements [19]. A study by Rafferty et al. [20] proposes that the interaction between collaboration and autonomy “suggests synergy rather than conflict”. In other words, autonomy can be complementary to team work and enhance collaboration by promoting collegial relationships between team members [18]. While some findings have pointed towards the potential for a positive association between collaboration and autonomy, researchers have also raised the issue of silos, where members of a team operate in separate and unconnected roles. This concept suggests a more profound form of detachment and autonomy between professions that goes beyond the boundaries around tasks. Thus, collaboration and autonomy have been suggested as complementary aspects that can enhance health service delivery although, in extreme forms the latter may inhibit team functioning.

Boundaries between professions on a team can form not only around interactions, but also around the distribution of responsibilities of different professionals. The construction of these boundaries in interprofessional settings may result in a separation of responsibilities or a decrease in formal role demarcations [21] (role blurring) between professions. Hall discusses the possibility that role blurring will occur because of overlapping competencies [21]. Role blurring is considered beneficial by some while others oppose it and link it to role strain and confusion [21]. For example, certain professionals on the team might believe that their role is being encroached upon and that their sense of professional identity is eroding [21,22]. Others may be overwhelmed because they are trying to do everything and are experiencing uncertainty about the limits of their responsibilities [8,21,22]. While some professionals may perceive role blurring as a threat, others may see an opportunity to expand their responsibilities or to make the team more flexible and responsive to its client population [21].

Research describing role distribution and interactions between team members can be complemented by knowledge about within-team dynamics and how these may contribute to shaping role boundaries. Different elements can influence how professional boundaries are constructed. At the micro-level (our level of analysis), these influences include *structural elements* (the characteristics of the workplace) such as workload [21,23] and physical space [24,25]; *interpersonal elements* (dynamics between team members) including leadership [26] and education [25]; and *individual attributes* (dynamics that individual practitioners bring to the interprofessional team) such as attitudes and values [4,6].

On different teams, certain influences may be more significant than others, leading to different manifestations of role distribution and interdependency between team members. The manner in which role boundaries are manifested may have implications for teams and their clients. Several authors have provided insights into the implications around collaborative endeavours and sharing of responsibilities for professionals and patients. These include easing workloads [23]; shorter wait times [27]; and continuity of care [28].

Although much of extant research looks at themes related to interprofessional collaboration, few studies have focused specifically on roles or proposed integrative models of role boundaries and influences on role construction. The reviewed literature, while mentioning phenomena such as role overlap [7] and role clarification [5] does not specifically consider the elements of role construction as a main focus. More research is needed to study methods of promoting collaboration in the workplace [17], to understand the complex relationship between collaboration and autonomy [20,29], and to further examine the implications of interprofessional collaboration for professionals and patients [30]. In addition, Cameron [18] advises that researchers should be seeking team members’ individual accounts and perceptions of professional boundaries in order to inform structural changes to the provision of health care services. Investigation into micro-level processes of boundary work can provide insights that may aid in improving interprofessional collaboration and the integration of roles [31]. In this study we help respond to these gaps by exploring how task roles are constructed on interprofessional teams. We consider the types of roles boundaries that are present, the influences on the construction of these boundaries, and the implications for practitioners and patients. In doing so, we provide an integrated overview of the elements of role construction rather than a detailed examination of one component over another. The following question guided this study:

How are roles constructed within interprofessional health care teams? More specifically, we ask: What types of role boundaries are present within an interprofessional team? What are the influences on the construction of roles and role boundaries?

## Methods

This research uses a holistic, comparative case study approach to explore the dynamics of role construction. Comparative case studies may generate more compelling evidence than single case studies because they allow for the analysis of patterns between cases and the derivation of more robust results [32,33]. Our case selection strategy was based on purposive sampling [34]. The two cases allowed us to generate rich information [34,35] for our study of role construction on interprofessional primary health care teams. We chose teams composed of multiple professions working together to deliver health services to patients so that we could collect data on the interactions and distribution of responsibilities between team members and in so doing, help respond to our research questions. Purposive sampling is also used to gather a diversity of opinions [36]. The selected health care teams offer similar services in primary health care but also have diverse characteristics allowing our findings to be extended across more than one case. These points of divergence include the origins of the two teams, the models of primary health care and the age of the teams. Both teams – located in different provinces in Canada but operating within similar regulatory frameworks - provide primary health care services including consultations, diabetes care, hypertension management and blood monitoring (INR reviews). These two cases also show similarities in the types of professions found on their teams for example, nurse practitioners (NPs), registered nurses (RNs), registered practical nurses (RPNs), dieticians, social workers and pharmacists, and in the size of the teams that were studied. As Eisenhardt suggests, the health care teams also have diverse characteristics, so that our findings could be extended across more than one type of team [32]. Team 1 transitioned from a group of independent physicians working in the same clinic to an interprofessional team model (Family Health Team). Team 2 is an NP-led team with physician consultants and was created specifically to respond to the underserviced primary health care needs of the community in which it is situated. Further, Team 2 is a recently-established team, which compares to Team 1 that has been in operation for several years.

### Data collection

Data was collected through interviews, observations and written documents. The interview protocol was designed as a standardized open-ended guide to allow for comparison and responsiveness to participants’ experiences. The questions explored roles (distribution, overlap, expansion), team member interactions, influences on role construction, and implications for practitioners and patients. Interviews were conducted with 13 members of each team which allowed us to sample from a wide range of professions (Table 1). Team 1 is a subset of a larger organization. Similar to Team 2, it includes a variety of professionals who collaborate – with varying intensities – in the delivery of health care services. A cross-section of professions was sampled through the help of the manager and through snowball technique. All members of Team 2 were interviewed. Non-participant observations of 2 team meetings at each site were recorded to learn more about roles and interprofessional collaboration among team members. These meetings were attended by most participants in the study and covered topics such as program updates, new initiatives (e.g. recruitment strategies and same-day appointments), reviews of, and modifications to, existing processes and protocols. Written documentation - organizational charts, meeting agendas (with supplementary information about projects), program templates and websites - provided additional background information on the origin, evolution, objectives and types of services for each team.

**Table 1:** List of interviews

**Occupation**	**Team 1**	**Team 2**
Clinical director	0	1
Manager	1	1
Nurse practitioner	3	3
Physician	3	0
Nurse (RN and RPN)	1	2
Pharmacist	1	1
Dietician	1	1
Social worker	0	1
Mental health counselor	1	0
Chiropodist	1	0
Laboratory technician	0	1
Administrative assistant	1	2
**Total**	**13**	**13**

### Data analysis

Interview transcripts were coded thematically using deductive codes from the research questions and literature review themes (e.g. blurred roles, leadership) and inductive codes that emerged from the data itself (e.g. recruitment process) [37]. Pattern codes (e.g. influences) were used to achieve higher levels of abstraction. Qualitative software (Atlas) was used to facilitate the coding process. Intra-case analyses involved detailed case study write-ups for each site and this step in the data analysis allowed the unique elements of each case to emerge before generalizations were made across the cases [32]. For example, not all of the influences on role construction were relevant for participants from both teams. The inter-case analysis generated patterns across the two cases and we developed a model to illustrate the dynamics of role construction that emerged from these findings. This process was iterative and involved refining our representation of the data several times. For instance, based on our literature review, we began the analysis by thinking about the construction of roles in terms of a ‘spectrum’ of blurred and siloed roles. After immersing ourselves iteratively in the data – by returning to the data and then refining our representation of the dynamics of role construction several times in a cyclical manner – we recognized that these concepts were more nuanced. Blurred roles link closely to the distribution of tasks and the interchangeable or differentiated repartition of responsibilities whereas, siloing indicates isolation of health professionals from each other and this concept touches on interactions between team members. This level of analysis allowed us to identify a range of influences and elucidate their importance in relation to boundaries around interactions and the distribution of responsibilities. Finally, emergent concepts were compared with extant literature to find out whether the former confirmed or disproved the latter, an important step in extending a body of knowledge [32].

### Transparency

In qualitative research, transparency involves making the study design and data interpretation explicit and replicable [36,38]. In keeping with this requirement, we have strived to clearly define our research assumptions and to provide precise information about the methods and data analysis. Another element of transparency is engaging in reflexivity around researcher assumptions and biases throughout the process and ensuring that the findings follow rigorously from the data [38]. The analysis was reviewed iteratively by two members of the research team to improve the thoroughness in interpreting the data. Also, a participant from each team reviewed the conceptual model and intra-case analysis for their research site. Both teams gave precision to the descriptive information and indicated that the elements presented in the case study were reliable. These member checks are a ‘crucial technique’ for establishing credibility [39]. Finally, triangulation of the data was achieved through the use of different data collection modes to establish converging lines of evidence [33].

Ethics approval was received from the University Ethics Board. Ethical issues considered included informed consent, anonymity and confidentiality of the teams and the participants. The project was presented to each team at the outset and participants provided written consent for the interviews and observations.

## Results

In this section, we start by presenting the comprehensive model that emerged from the analysis, and then move to a presentation of each of the elements of the model.

**Figure 1:**
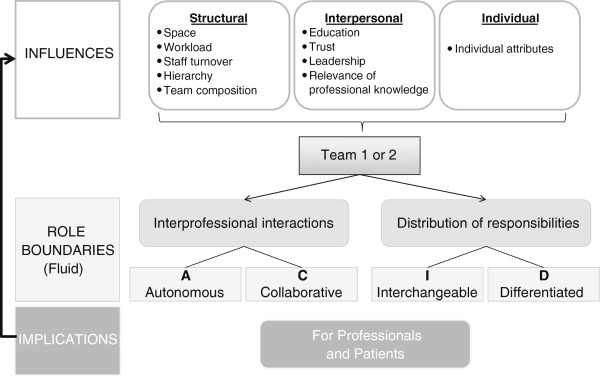
**Model of role construction.** This figure represents the elements of role construction that emerged from the analysis including the types of role boundaries, the within-team dynamics that influence these boundaries, and the implications of role boundaries for professionals and patients.

The model is presented in Figure 1. Based on the findings from our study and on the literature on professional boundaries and collaboration, we categorized roles along two dimensions - as autonomous or collaborative, and as interchangeable or differentiated. The autonomous-collaborative category of role boundaries refers to the manifestation of interprofessional interactions on a team; less interaction generally implies more autonomy. We use the term ‘differentiation’ to indicate the delineation of team members’ responsibilities and ‘interchangeability’, where one profession performs some of the same tasks as another. Situated in the middle of the diagram are the boundaries that form around interprofessional interactions (collaborative and autonomous roles) and the distribution of professional responsibilities (interchangeable and differentiated roles). These are four ideal types of roles and there is fluidity around how the boundaries of roles are constructed and re-constructed. At the top of the diagram are the influences that shape the boundaries around roles as per our findings. The variable nature of these different types of influences and the team context means that the former do not always manifest themselves in a pre-determined fashion (e.g. staff turnover could be high or low). These influences are not mutually exclusive and can affect each other. For example, rapid staff turnover makes it difficult for team members to build trust between each other. As indicated at the bottom of the diagram, the construction of role boundaries on an interprofessional team also has implications for team members and patients. Role construction is a dynamic process: the arrow from ‘implications’ to ‘influences’ illustrates a feedback loop.

### Role boundaries

*Collaborative* roles occur where team members have frequent interactions and knowledge exchanges; *autonomous* roles occur where team members have fewer interactions, less collaboration and work more independently from each other (note that these autonomous roles still have the potential to be complementary to the team).

“I really rely heavily on the other people that work here…I often share my findings on a patient with other colleagues or they share with me.” Team 2, NP - Participant 4 (collaborative)

“I’m with patients all day…I’m not going to sit there and talk about work ever with anybody really…I’m more independent.” Team 1, Chiropodist - Participant 13 (autonomous)

*Interchangeable* roles arise where the responsibilities of different team members overlap and they can be beneficial for example, by helping to ease the workload of another health professional. *Differentiated* roles occur when team members have separate and distinct responsibilities.

“[New patients] fill out an intake form…Once that is filled out, we have them book an intake appointment where they may meet with the RN, RPN or the NP, depending on availability, where the intake is reviewed. Team 2, Clinical director - Participant 1 (interchangeable)

“NPs have a larger practice… They can prescribe, they can make a diagnosis, we (RNs) cannot.” Team 1, RN - Participant 9 (differentiated)

### Influences

In this section, we discuss structural, interpersonal and individual influences on role construction and boundaries by attending to impacts on interaction and distribution of responsibilities.

#### *Structural influences*

Structural influences refer to the characteristics of the workplace and include physical space, workloads, turnover, hierarchy and team composition. *Physical space* is an influence that is often mentioned by participants from Team 1 yet is rarely seen to have a direct effect by Team 2. Some health professionals will be located in proximity to one another while others will be farther apart. The layout of clinical space can affect the potential for interactions among team members. On Team 2 physical space may be perceived to have a smaller impact because team members are at close distance to each other and therefore see one another frequently. During visits with Team 1, we observed that team members were located on different floors and buildings whereas almost all members of Team 2 had offices in the same hallway. The interview data supports these observations: many Team 1 participants commented that their physical distance from other team members had an impact on their ability to interact with each other whereas members of Team 2 did not address the issue of physical distance. It is possible that physical space is seen as an influence by team members when it prevents interactions whereas, the positive impacts of clinical space may be accepted as the status quo.

On Team 1, *workloads* influence role construction both in terms of the frequency of interactions between health professionals and the distribution of responsibilities. For example, a heavy workload can be a factor in reducing opportunities for interaction with others. Also, the presence of long waiting lists for patients to schedule appointments with some allied health professionals tends to create a situation where NPs and physicians offer some services that are in the area of expertise of another health professional so that patients can avoid the wait. This strategy also alleviates some of the work pressure on the allied health professional in question.

“The chiropodist is specialized in foot care, wounds, warts, injuries, nails…But we [NPs] still do wart treatments. So there is a duplication of services provided but she can’t see everyone: she’s already got a ridiculously long waiting list.” Team 1, NP - Participant 8

Team 2 appears to have some buffering characteristics that facilitate interaction in spite of workload. All Team 2 members are located in proximity to each other which allows for frequent informal meetings as team members walk up and down the hallway. Also, Team 2 has more intensely scheduled team interactions (e.g. a two-hour team meeting every month vs. a one-hour team meeting every two months for Team 1). Similarly to Team 1, the workload on Team 2 tends to affect how responsibilities are spread out among team members: the amount of work to be accomplished can compel NPs to retain responsibilities or to delegate more to others so that they can focus on the areas of their expertise that do not overlap with other team members.

*Turnover,* similar to physical space, is seen to have an influence by Team 1 but not by Team 2 participants. In comparing the two cases, it can be noted that Team 2 is quite young – at the time of interviews, many of the team members had not yet experienced the effects of staff turnover on this team – therefore it is unlikely that current team members have a good indication of how and if turnover affects their role construction in this context. On Team 1, participants commented on the high turnover among some professions which appears to affect role boundaries in terms of team members’ ability to develop collegial relationships and collaborate. It also influences whether primary care providers choose to access chronic disease management programs and services, an aspect that can change the primary care providers’ responsibilities towards the patient.

On both teams, physicians and/or NPs are at the top of the *hierarchy* and chain of responsibility for the patient and have the power to refer and delegate responsibilities. They can influence the types of responsibilities associated with other roles on the team and also the extent to which they collaborate and are interrelated with other health professionals. For example, some physicians were found to use their status to facilitate the professional development and growth of the NP and other allied health professional roles on Team 1.

“There are definitely power dynamics. I think in general physicians tend to hold more weight.” Team 1, Mental health counselor - Participant 12

Positional power may be used to influence roles positively or negatively. The following example illustrates how team members higher in the hierarchy may exert influence over the roles and interactions with other professions:

“One of the interesting things that we found and worked through was the whole ‘grabbing-and-letting-go’ process because there are a lot of similar tasks in the roles [of NPs and RNs]…How we worked to build a process was to keep reminding the nurse practitioners in this model that you are now similar to a family doctor and those RNs are similar to the nurse practitioners. So you know exactly what it’s like to feel like you’re compressed and not working your full scope, why would you do the same to an RN? And when they start thinking like that and putting themselves in that position, that’s when they start working together and they learn how to truly work as colleagues.” Team 2, Manager - Participant 2

In addition, a team member’s responsibilities can change as a function of the professional *composition* of the team. When the professional composition changes, the responsibility for certain tasks might shift as well. For example, adaptation may take place because a new team member has more knowledge in an existing area of health service delivery. Different combinations of professions on the team, including the types of professions and number of work hours, results in different interactions and distributions of responsibilities among the team members.

#### *Interpersonal influences*

Interpersonal dynamics are the dynamics between team members and include such elements as professionals’ education and understanding of each other’s roles, trust, leadership, and consultation of each other based on the relevance of professional knowledge. *Education* is an important influence because health professionals will not necessarily join the team with an understanding of the responsibilities of all the other professions and how to engage these professionals’ services in the care of the patient. For both teams, education influences autonomous-collaborative role boundaries.

“As part of my orientation when I started working here, I spent some time with each of those disciplines to get a little bit clearer idea of what their role is. I think it enhances the team, it enhances my work.” Team 2, NP - Participant 5

*Trust* is a relational factor that affects the extent to which professionals are collaborative and are willing to delegate and share responsibilities. On both teams, providers develop trust by interacting with colleagues on a professional and personal level. The presence of trust makes providers feel more comfortable in relying on each other’s expertise and can foster greater sharing of responsibilities.

“If you’ve got a clinical pharmacist who is very approachable, demonstrates to the physicians and NPs…that she’s very knowledgeable…, answers their questions in a very helpful manner, provides good advice to them and to their patients, then people consult the pharmacist and the things they ask the pharmacist to do on behalf of their patient continue to increase.” Team 1, Manager - Participant 1

*Leadership* can influence the distribution of responsibilities and foster the collaborative tendencies of the team. Staff from both teams readily identified with formal leadership (e.g. clinical director and team manager). At this level, leaders can be key in helping to integrate new professionals into the team and creating a sense of team belonging.

“We all have a say in the hiring of our teammates and we discuss roles ahead of time. We collaboratively get together and say ‘okay, what are we missing in our model of care and which position would be able to fill that void’.” Team 2, Manager - Participant 2

Leadership can also facilitate opportunities for interprofessional interactions through formalized events such as team meetings. Leaders can contribute to making these meetings a space for team members to initiate new opportunities for team collaboration. For example, during a Team 1 meeting, we observed a nurse practitioner bringing forward her idea for a new ‘internal education day’ where team members would present and teach each other about different clinical topics. The leaders facilitated this endeavour by including it on the agenda, introducing the item in a supportive manner and ensuring that a date and presenter were chosen for the first education day. Formal leaders on Teams 1 and 2 are active in empowering staff to grow in their roles and in giving them more autonomy to pursue clinical areas of interest to them.

Team members consult some colleagues for advice and expertise more regularly than others. The *relevance of professional knowledge* is an influence on collaborative role boundaries because health professionals tend to collaborate more frequently with the professions that can provide them with additional knowledge and information to inform their care decisions and vice versa.

“I really rely on the pharmacist to ensure…I’m using the optimal medication for an individual patient…so I interact with him a lot. I do interact with the dietician and social worker as well, but it’s more…on a nice-to-know type of basis rather than really relying on the skill set of another professional to help me in my role.” Team 2, NP - Participant 4

For Team 1 and Team 2, the relevance of professional knowledge and expertise can have an impact on the frequency of interaction between different professions on the team. This dynamic may contribute to the construction of more autonomous or more collaborative role boundaries.

#### *Individual influences*

*Individual attributes*, such as an individual’s approach to care or perspective on interaction with other team members, can be a factor in determining how much team members are willing to work and grow in their role collaboratively (in addition to having autonomy) and can also affect the distribution of responsibilities.

“Some providers feel that as primary care provider they should be providing all of the primary care and doing everything and they really don’t refer a lot. But they might use my services in another way: drug information questions. So for some physicians I’m really exclusively a drug information pharmacist. For other physicians, I’m much more involved in a collaborative care approach where they’ll refer me a patient and ‘can you recommend what should I do about this’ and in those instances it’s collaborative and I’m a part of patient care, whereas with others it’s very separate.” Team 1, Participant 11 – Pharmacist

Some providers may see the patient as the ‘team’s patient’ and this view can influence them to delegate more responsibilities and to collaborate with other health professionals. Others see the patient ultimately as their responsibility and may feel uncomfortable in relying on other team members to provide care for their patients. Also, individual traits such as timidity and confidence can influence team members’ integration within an interprofessional team and their interaction with other health professionals.

### Implications for professionals and patients

In terms of the implications stemming from autonomous-collaborative role boundaries, both Team 1 and 2 participants find an advantage in being *supported* by the knowledge of other professions (for example, physicians and NPs see consultations with a pharmacist as valuable encounters) and link collaborative exchanges with *professional satisfaction*. Some professionals also expressed that they gained satisfaction from the autonomous dimensions of their role.

“I love having our pharmacist here…the NP. They’re great supports and it’s nice feeling that you’re not alone in taking care of a huge number of people. That there is a safety net. It’s nice to know that there are other people you can call and say I’ve got to change her off of all of these medications, can you please help me here.” Team 1, Physician - Participant 4

“I think if I were on [another team], I would not be seeing every patient; I would be perhaps seeing more routine types of patients; and then there would be these doctors asking me about my patient care all the time, and questioning me on that. And here we don’t have that. So being given that autonomy, being given that responsibility for me is just to work extra hard to do a really good job, and that’s what I strive for every day…It’s like a breath of fresh air, compared to anywhere else I’ve ever worked. Even though they all say it’s a team, it’s not a team compared to here. I can’t stress that enough; this is amazing here in that way.” Team 2, Participant 5 – NP

Participants mentioned that patients can benefit from collaborative endeavours by receiving more *holistic care* and through better coordination and *continuity* of health services. Turning to the implications of interchangeable-differentiated role boundaries, interviewees on both teams commented that the interchangeable nature of some responsibilities contributes to *alleviating the burden of their workload*.

“With the ‘Well-Baby’ visits…I usually do immunizations but, if I’ve got two to give, then sometimes they [NPs] will come in and help me, even though that’s really not their role. But it’s still within their scope…so they will come and help me do it.” Team 2, RN - Participant 6

Nevertheless, overlapping responsibilities can also engender *confusion around roles*: this situation is experienced by members of Team 1 but was not a salient issue for Team 2. Possible explanations for the variability in this implication may include that Team 2 has more opportunities for addressing misunderstandings. Team 2 holds team meetings more frequently than Team 1 which may make it easier to facilitate a standardized understanding of roles and responsibilities and clarify procedures and explanations. For instance, during one of Team 2’s meetings, the clinical director shared an experience with miscommunication at the clinic and solicited input for creating better risk management for verifying pharmaceutical information on new patients’ intake forms. Team members discussed various ideas around how to modify the oversight of pharmaceutical information. With the entire team present and participating, the challenge was addressed, a new protocol for verifying drug information was agreed upon and revised responsibilities for the pharmacist were elaborated. On Team 1, the staff meetings happened less frequently and discussions tended to remain at the level of administrative issues and program updates. The manager chairing the meetings commented several times on the need to attend to items quickly and efficiently because of time constraints. Team 2 appears to have more opportunities for interactions to raise issues and refine shared understandings of responsibilities and areas of expertise.

Team members from both cases suggest that the differentiation of roles can entail certain advantages such as allowing the skills and abilities of professionals to be focused on a specific area of expertise within the team (*maximization of skills*) and decreasing the likelihood of *power struggles* related to overlapping responsibilities.

“I don’t know if I think anybody has any more power. I work really collaboratively with most of the NPs. I don’t feel like I could do their role and I don’t think that they really feel that they could do mine. So I think that we respect each others’ boundaries and limits.” Team 1, Pharmacist - Participant 8

Similarly to the way in which the interchangeability of responsibilities can ease the workload of a health care provider, it was also found to result in *shorter wait times* for patients of both teams. *Greater familiarity with the whole care team*, due to the interchangeability of responsibilities, is also an advantage suggested by Team 2.

## Discussion

The analysis explored themes emerging from the data and proposed a model to conceptualize the elements of role construction including the different types of boundaries around roles, influences on the construction of role boundaries (structural, interpersonal, individual) and the implications of role construction for professionals and patients. Our comparative approach allowed us to identify the similarities and differences between the two cases and to substantiate them with findings from our data. The findings indicated that most of the influences and implications are similar across the two cases, although there is some variability as we highlighted in the Results section. In this section, we compare our findings to extant literature.

### Role boundaries

The definition of role construction – the creation and negotiation of taskwork – aligns well with the two categories of role boundaries that we developed from the data in this study. Taskwork, a notion introduced in our literature review, involves both carrying out responsibilities associated with group goals and supporting other team members’ efforts to do the same [11]. Similarly, the categories of role boundaries that we derived are grouped around the distribution of responsibilities and around interactions between team members. The contours of these boundaries are shifted and re-shaped by various influences, of which we considered several that are present at the team or micro- level. We attended to the interactional dimension between team members as an element in the construction of different roles in a team environment. Some team members had fewer interactions and were more autonomous while others had more interactions and were more collaborative. Also, some health professionals interacted frequently with particular team members and very little with others. To look at interactions in the context of interprofessional roles, we explored the manifestations of autonomous-collaborative role boundaries for the two cases.

The implications of autonomous-collaborative role boundaries in this study suggest that autonomy may be an important element of team functioning. Though this statement may seem counter-intuitive, empowering team members to develop autonomy can enhance collaborative interactions. As one NP on Team 2 indicated, being given autonomy motivates her to work harder and contributes to her sense that she is part of a team. Some researchers have found that collaboration is an interpersonal process [17] and requires joint involvement (two or more parties) in intellectual activities [3]. If one of these parties has limited ability to accomplish some tasks independently or autonomously, then they may be less able to make meaningful contributions to discussions with others around patient care. Ensuring that all providers have an appropriate level of autonomy is in one sense allowing health professionals the respect of their profession and their knowledge within the team. A certain amount of autonomy may enhance participation and make a role more meaningful and rewarding. The synergy between collaboration and autonomy is supported by the work of Rafferty et al. [20] and Maylone et al. [29] who state that autonomy can contribute to more effective teamwork. Our study extends understanding of the relationship between autonomy and collaboration on health care teams by highlighting the patterns of interaction between team members. In addition, the findings uncover a level of complexity by suggesting that interactions are not equal between all team members but vary according to different influences such as the relevance of professional knowledge. It would be useful to explore autonomy in greater depth to understand when it can be a constraining or facilitating factor in interprofessional teams.

D’Amour et al. have indicated that sharing responsibilities is a collaborative endeavour [3]. In the context of studying interprofessional roles, we have made a distinction between the concept of autonomous-collaborative relationships (interactions and knowledge exchanges) and the distribution of responsibilities among team members. We made this distinction because we found that team members could be autonomous (limited contact with others) and still have interchangeable responsibilities with other professions. In a synthesis of research on interprofessional teams, Virani [40] states that “team members divide the work based on their scope of practice.” We have sought to extend this understanding by further examining the micro-level complexities around the distribution of responsibilities and by proposing to view it in terms of interchangeable and differentiated role boundaries. In this way, we are able to elaborate what, in addition to scope of practice, may be influencing the distribution of responsibilities on an interprofessional primary health care team.

Furthermore, different types of role boundaries can have conflicting implications. For instance, while more interchangeable roles could help to lessen the workloads of team members, they could also increase the potential for power struggles because the roles of various professions would become less differentiated. For example, the manager on Team 2 observed that the overlap between the roles of NPs and RNs on the team initially required interventions with NPs to convince them to relinquish control and share certain responsibilities. In the beginning, the overlap created some friction on the team and the subsequent adjustment helped to improve the collegial atmosphere between the team members. Similarly, a study on integrating pharmacists into general practice found that pharmacists’ ‘value-added’ services appeared less threatening to family physicians than responsibilities that duplicated the role of the physician [41].

A comparison of the implications of role boundaries for Team 1 and 2 gave similar indications that advantages accompany both the interchangeability and the differentiation of responsibilities (as well as autonomous and collaborative interactions). This finding suggests that tending to the extreme of one type of role boundary or the other may engender negative implications. For example, team members who do not feel that they have autonomy may be dissatisfied with their role on the team. Therefore, it will be important to be aware of the implications of different role boundaries and to strike a balance between autonomous and collaborative roles, and between interchangeable and differentiated roles.

### Influences

The challenge of carving out professional territories on health care teams is an issue raised by D’Amour et al. [3] in their literature review on interprofessional collaboration and noted in our introduction to this paper. We have presented a range of elements within the two teams under study that may affect how boundaries are drawn up and shared between team members. Several of the influences on role construction that we identified parallel the literature on health care teams. Salient factors for team functioning and effectiveness that we have proposed to have an impact on role boundaries include: physical space [42], workload [23], staff turnover [43], hierarchy [44], team composition [24], education [45], leadership [46], trust [47] and individual attributes [4]. The inter-case analysis of Team 1 and 2 yielded insight into the salience of some influences (e.g. the perceived significance of turnover for role boundaries was influenced by the age of the team). Many dynamics and contextual factors that affect interprofessional collaboration have been discussed in the literature. Our examination of role construction offers findings about how some of these dynamics may influence autonomy and the distribution of responsibilities as well as collaboration in the context of primary health care teams. This study provides further insight into how the significance of some of these influences may vary between teams. In addition, although several influences examined in our study have been addressed in the literature, we contribute a comprehensive model that groups these influences under broader multilevel categories, and we bring further nuance to several of these elements. For instance, authors have illustrated the tendency for hierarchical relationships to hinder collaboration between professions [6]. While we saw evidence of this phenomenon in our study, we also found that positional power can be positively used to develop the responsibilities of, and interactions with, other team members.

As another example, the relevance of professional knowledge was not considered as an influence in the literature reviewed, however it appears to contribute to the frequency of interactions between different professions. Our data showed the relevance of professional knowledge to be an important influence on autonomous-collaborative role boundaries. It helps to predict which professionals will interact more often with each other. It is logical that team members will engage in more knowledge exchanges with health professionals whose expertise is more relevant to their own responsibilities. This suggests that team members may form patterns of interaction based on the relevance of professional knowledge. It also indicates that the intensity of collaboration is not the same between all team members.

This influence could be important for the co-location of team members. For example, since physicians and NPs find the knowledge of the pharmacists to be very relevant to their care decisions and consult them frequently, the co-location of these health professions may facilitate this interaction. Oandasan et al. [42] examined the impact of space in interprofessional primary health care teams and found that physically separate workspaces produce barriers to working directly with other team members whereas co-location enhances visibility and access and creates opportunities for informal interactions. Despite the advantages of these spatial arrangements, co-location is not always desirable: small shared spaces and offices can lead to feelings of being crowded and having one’s privacy invaded [42]. Given that most health care teams are faced with unique spatial arrangements and challenges, taking the relevance of professional knowledge into account may help to inform decisions around how to co-locate team members and arrange the spaces that are available to each team.

In terms of individual influences, our findings support and extend the literature on the impact of individual attributes on interprofessional collaboration [26]. Our case studies show that individual dynamics influence the delegation of tasks and the frequency of interprofessional interactions. Despite the fact that the patients in one case study are registered directly to a physician whereas the patients in the other case study are registered to the clinic, primary care providers in both cases had mixed perceptions of a patient’s ‘belonging’. The view that a patient is ‘ours’ instead of ‘mine’ seems to encourage more interchangeability of responsibilities (where applicable) and also more interactions and knowledge exchanges to inform patient care.

Individual traits also influence the ability of team members to work in a team environment. In a study looking at transitions to the Family Health Team model in primary health care, Ragaz et al. found that some clinical and administrative team members left their team because of inflexibility and discomfort with change [48]. These authors cited attitude as the ‘most important hiring criterion’ [48]. Similarly, in our study, there were team members who were more or less at ease in collaborating and sharing responsibilities with other professions. One pharmacist in our study remarked that different physicians’ approaches to care and interaction with other team members affected her level of involvement in health services delivery (from researching pharmaceutical information to joint involvement in adjusting patient medications). These findings support the literature suggesting that individual attributes have an impact on team work. Health care teams that are in the process of recruitment should therefore consider not only the clinical experience of a candidate but also how their individual characteristics will allow them to fit in with the rest of the team members.

### Implications for practice

The model and findings presented in this study may add to interprofessional health care teams’ appreciation of the importance of role construction for health professionals and patients. More specifically, it calls for professionals and those in leadership positions to be aware of the need to develop autonomous and collaborative aspects of roles. Likewise, it is necessary to recognize the potential benefits of both interchangeable and differentiated dimensions of roles on the team. Attention should be given to the contextual factors of a specific team that may influence role construction: teams should consider whether they need to adapt their current strategies in order to modify either of these two aspects of role boundaries.

### Limitations

Similar to other case study research, the findings of this study are derived from the two cases that were the subject of our analysis and generalizability to other settings may be constrained [32]. Nevertheless, the contextual descriptions facilitate the transferability of the findings to other settings with similar contexts [49]. Our study targeted primary health care teams. The findings can be transferred to teams that operate in similar contexts, such as community mental health care teams where psychiatrists, psychologists, social workers, nurses and other professionals work together to deliver services. It should be noted that these teams tend to operate in non-crisis driven environments, unlike hospital-based interprofessional emergency teams for example. Further research should allow the exploration of role dynamics and influences on teams operating in different contexts. In addition, these findings offer a high level perspective on the dynamics of role construction and our model cannot capture all of the complexities associated with these concepts. It would be important to seek additional insights about the meaning of role boundaries for members of health care teams. Also, to gain a deeper understanding of role boundaries it would be helpful to learn more about how the way in which boundaries are shifted and shaped within teams differs from how they can be influenced externally. Future studies may want to engage at length with one or more concepts in our model to provide further theoretical and empirical analysis of role construction.

## Conclusions

Interprofessional primary health care teams may use this research to enrich their knowledge of the elements of role construction. It may also provide them with an exploratory model through which they can examine the dynamics of role construction in their own setting. It would be helpful to learn more about how to utilize various influences from the outset of an interprofessional team in order to facilitate role construction that will have positive implications for professionals and patients. Our study provides insights and avenues to be considered by managers and researchers interested in interprofessional arrangements in health care.

## Abbreviations

NP: Nurse practitioner; RN: Registered nurse; RPN: Registered practical nurse.

## Competing interests

The authors declare that they have no competing interests.

## Authors’ contributions

KM developed the study design, collected the data, carried out the data analysis and drafted the original manuscript. SC and IB contributed to the conceptualization and design of the study. SC helped in guiding the analysis and interpreting the findings. All authors contributed to the literature review and read and approved the final manuscript.

## Pre-publication history

The pre-publication history for this paper can be accessed here:

http://www.biomedcentral.com/1472-6963/13/486/prepub
